# A high therapeutic efficacy of polymeric prodrug nano-assembly for a combination of photodynamic therapy and chemotherapy

**DOI:** 10.1038/s42003-018-0204-6

**Published:** 2018-11-21

**Authors:** Xiaoqing Yi, Jun Dai, Yingyan Han, Min Xu, Xiaojin Zhang, Shijie Zhen, Zujin Zhao, Xiaoding Lou, Fan Xia

**Affiliations:** 1 Engineering Research Center of Nano-Geomaterials of Ministry of Education, Faculty of Materials Science and Chemistry, China University of Geosciences, Wuhan, 430074 China; 2 Department of Obstetrics and Gynecology, Tongji Hospital, Tongji Medical College, Hubei Key Laboratory of Bioinorganic Chemistry & Materia Medica, School of Chemistry and Chemical Engineering, Huazhong University of Science and Technology, Wuhan, 430074 China; 3 Center for Aggregation-Induced Emission, State Key Laboratory of Luminescent Materials and Devices, South China University of Technology, Guangzhou, 510640 China

## Abstract

Combination of photodynamic therapy and chemotherapy has been emerging as a new strategy for cancer treatment. Conventional photosensitizer tends to aggregate in aqueous media, which causes fluorescence quenching, reduces reactive oxygen species (ROS) production, and limits its clinical application to photodynamic therapy. Traditional nanoparticle drug delivery system for chemotherapy also has its disadvantages, such as low drug loading content, drug leakage, and off-target toxicity for normal tissues. Here, we developed a reduction-sensitive co-delivery micelles TB@PMP for combinational therapy, which composed of entrapping a red aggregation-induced emission fluorogen (AIEgen) for photodynamic therapy and PMP that contains a reduction-sensitive paclitaxel polymeric prodrug for chemotherapy. AIEgen photosensitizer illustrates a much improved photostability and ROS production efficiency in aggregate state and PMP loads a high dose of paclitaxel and carries a smart stimuli-triggered drug release property. This co-delivery system provides a better option that replaces AIEgen photosensitizer for cancer diagnosis and therapy.

## Abstract (web summary)

Xiaoqing Yi et al. report a co-drug delivery micelle system that demonstrates a high therapeutic efficacy for cancer. This system shows a much improved drug load, photostability, and production of reactive oxygen species, compared to traditional photosensitizer-loaded nanoparticles.

## Introduction

Photodynamic therapy (PDT) plays a crucial role in the treatment of cancer in the clinic, which can improve the quality of life and median survival of patient with tunable phototoxicity and minimal invasion^1–4^. PDT utilizes the photosensitizer, light irradiation and surrounding dissolved oxygen, to produce reactive oxygen species (ROS), and subsequently causes apoptosis or necrosis of treated cells^5–7^. Therefore, the effect of PDT could be accurately controlled by regulating the time and intensity of light exposure^8,9^. Nevertheless, the overall PDT performance is yet unsatisfactory. One reason is that lower treatment efficacy would appear due to oxygen consumption during PDT process especially in hypoxic solid tumor environment^10–12^. Also, chemotherapy is an important clinical treatment strategy after surgical resection of primary solid tumors^13^. However, the effect of chemotherapy is still limited due to emergence of multidrug resistance, systemic toxicity by non-specific drug distribution, poor water solubility, and rapid clearance by the reticuloendothelial system during the treatment^14^. Combination of PDT and chemotherapy, thus, has been emerging as a new strategy for cancer treatment such as solid tumors^15,16^. For PDT, ROS will damage vascular endothelial cells and induce the formation of endothelial intercellular gaps, causing leaky tumor microvasculature and improved enhanced permeability and retention (EPR) effect^16^. Based on this, PDT and chemotherapy can compensate for each other’s weaknesses (easy aggregation of photosensitizer, drug leakage) to enhance the treatment effect to a certain extent. What is more, both vascular and cellular effects contributed to PDT and chemotherapy efficacy can be affected by drug-light interval^17^. However, the intrinsic shortcomings of conventional PDT and chemotherapy, respectively, are not eliminated in fact^18–20^.

For PDT, due to the π–π stacking and rigid planar structures, traditional photosensitizer, such as porphyrin and its derivatives, could naturally aggregate in aqueous media, and result in quenched fluorescence and reduced ROS production efficiency due to aggregation-caused quenching (ACQ) effect^21^. It means that there is only weak or even no emission as well as poor ROS production in high concentration or aggregate state of traditional photosensitizer. Fortunately, aggregation-induced emission fluorogens (AIEgens) provide an efficient approach to overcome the ACQ problem. In 2001, Tang’s group discovered a special fluorogen which is non-emissive in solution state but could emit high fluorescent efficiency in aggregate state due to the restriction of intramolecular rotation^22^. This feature has been used to develop specific light-up probes^23–25^ and AIE nanoparticles for evaluation of image-guided tumor resection^26^, cell tracking^27^, visualization of drug delivery processes^28^, imaging of cancer cell progression, and continuous monitoring of biological processes^29^. Besides, several AIEgens have also been developed to serve as effective photosensitizer for PDT. Compared to the traditional photosensitizer, one advantages of AIEgen photosensitizer is that there is still a strong bright emission and high ROS production efficiency in the state of aggregation. Recently, AIEgen photosensitizer have been developed for chemiluminescence-guided PDT anticancer therapy and cancer cell ablation in vitro and in vivo^30,31^.

For chemotherapy, as promising anticancer drug carriers, biocompatible and biodegradable amphiphilic polymer micelles-based drug delivery system has been widely employed in the past ten years^32–35^. It can overcome the disadvantage of high hydrophobic drugs, improve the bioavailability of drugs and elongate blood circulation time^36,37^. As a result, micelles-based drug delivery system shows selective tumor targeting property by EPR effect. Polymeric prodrugs that chemically conjugate the amphiphilic polymer with chemotherapeutics drugs have already entered clinical trials^38,39^. Self-assembly polymeric prodrug possess some potential advantages, such as high drug loading content and enhanced chemical stability, which could also be used as carriers to load another drug for combinational therapy^40,41^. Moreover, premature chemotherapeutic release is suppressed by chemically conjugating the amphiphilic polymer with chemotherapeutics drugs to form stimuli-responsive polymeric prodrug^41–43^. That is, undesired leakage of the drugs during circulation could be reduced. At the same time, side effects in normal tissues will be restrained effectively. Furthermore, the controlled drug release from stimuli-responsive polymeric prodrug micelles in desired time and space could be achieved by sensing the difference between intracellular and extracellular tumor microenvironment, such as the redox potential caused by a great difference in the concentration of glutathione (GSH) between extracellular (2–20 × 10^−6^ M) and intracellular (2–10 × 10^−3^ M) environment of cancer cells^32,37,44^.

**Fig. 1:**
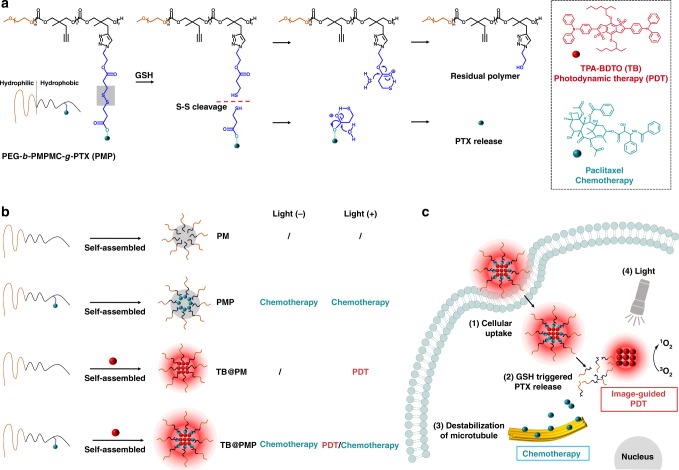
Polymeric prodrug nano-assembly entrapping AIEgen for combinational image-guided PDT/chemotherapy. **a** Reduction-sensitive paclitaxel prodrug (azido-SS-PTX) was grafted onto amphiphilic polymer by azide-alkyne click reaction to give a reduction-sensitive polymeric prodrug, poly(ethylene glycol)-*b*-poly(5-mthyl-5-propargyl-1,3-dioxan-2-one)-*g*-paclitaxel, PEG-*b*-PMPMC-*g*-PTX (PMP). The release mechanism of free paclitaxel from PMP can be ascribed to high intracellular concentrations of reducing agents, such as glutathione. **b** Amphiphilic polymer could self-assemble into micelles, and then encapsulate hydrophobic AIEgen into the core of micelles by hydrophobic effect. PM, PMP, TB@PM, and TB@PMP micelles were prepared by dialysis in aqueous solution, respectively. PMP (−), PMP (+), TB@PMP (−) can be used as chemotherapy groups, and TB@PM (+) can be used as PDT group. TB@PMP (+) was employed for combinational PDT/chemotherapy, while PM (−), PM (+), TB@PM (−) were employed for negative control. **c** TB@PMP micelles internalization and therapy process. Reduction-sensitive co-delivery system TB@PMP could targets tumor interstitial fluid in a passive manner via the EPR effect. After cleavage of disulfide bonds under high concentration of GSH in tumor cells, free paclitaxel was released to disrupt the microtubule. Meanwhile, TB generate cytotoxic ROS to damage the tumor cell under light irradiation

In this work, a reduction-sensitive co-delivery system based on polymeric prodrug poly(ethylene glycol)-*b*-poly(5-mthyl-5-propargyl-1,3-dioxan-2-one)-*g*-paclitaxel, PEG-*b*-PMPMC-*g*-PTX (PMP), entrapping a red emissive AIEgen photosensitizer (TPA-BDTO, TB) was developed for combinational image-guided PDT/chemotherapy. As shown in Fig. 1a, the chemotherapy drug paclitaxel was grafted onto the amphiphilic polymer backbone via disulfide bond to give the reduction-sensitive polymeric prodrug PMP. The amphiphilic polymeric prodrug PMP could self-assemble into micelles in aqueous solution and then entrap the red emissive AIEgen photosensitizer (TB) through hydrophobic effect to prepare TB@PMP micelles. The biocompatibility and circulation time in the blood stream of TB@PMP micelles would be improved by the hydrophilic shell layer of PEG. As a comparison, different micelles including PM, PMP, TB@PM were synthesized and employed for control groups (Fig. 1b). When intravenously injected into tumor-bearing mouse, the nanosized TB@PMP micelles were enriched in tumor interstitial fluid by a passive manner via EPR effect. After the TB@PMP micelles were uptaken by the tumor cells, the disulfide bond in PMP was cleaved because of high concentration of GSH in tumor cells, which induced the breakdown of the neighboring ester bond to generate native paclitaxel in tumor cells. The released paclitaxel binds to a specific site of tubulin to prevent its depolymerization (Fig. 1c). As a result, the balance between microtubule aggregation and deaggregation is disrupted, causing the failed replication and eventually leading to cancer cell apoptosis. Meanwhile, the TB could generate cytotoxic ROS to damage the tumor cell under light irradiation. Therefore, the co-delivery system TB@PMP was developed for combinational cancer therapy. It does not suffer from the above-mentioned drawbacks, illustrating distinct improved effects both in vitro and in vivo and provides a better option that replaces AIEgen photosensitizer in cancer diagnosis and therapy.

## Results

### Synthesis and characterization of PM, PMP, TB@PM, and TB@PMP micelles

**Fig. 2:**
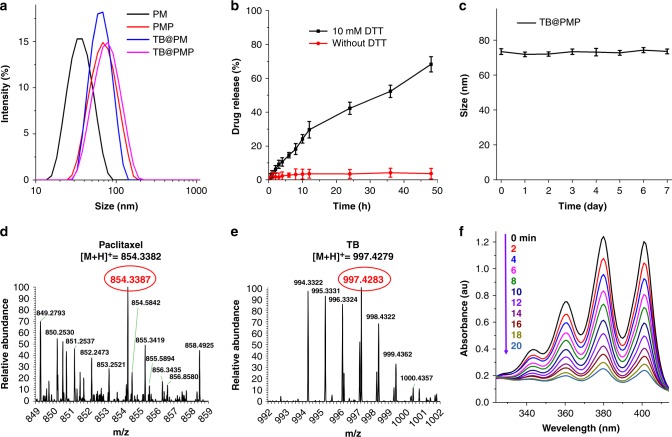
Synthesis and characterization of PM, PMP, TB@PM, and TB@PMP micelles. **a** Hydrodynamic size distribution of PM, PMP, TB@PM, and TB@PMP micelles in PBS. **b** In vitro release of paclitaxel from PMP micelles in PBS (pH 7.4, 0.1 M) containing 0.1% (w/v) Tween 80 at 37 ℃ with 10 mM DTT or without DTT. **c** Stability assay of TB@PMP micelles in PBS during 7-day storage at room temperature. **d**, **e** Mass spectrum of the released products from TB@PMP micelles with the treatment of 10 mM DTT for 12 h. HRMS (paclitaxel): [M+H]^+^ 854.3387 (calcd 854.3382); TB: [M+H]^+^ 997.4283 (calcd 997.4279). **f** UV–vis absorption spectrum of the ROS indicator 9,10-anthracenediylbis(methylene)dimalonic acid mixed with TB@PMP micelles with 10 mM DTT incubated at 37 °C for 0.5 h (mimics intracellular reducing conditions of tumor cells) upon light irradiation (white light, 100 mW cm^−2^)

The reduction-sensitive TB@PMP micelles co-deliver paclitaxel and AIEgen photosensitizer combinational image-guided PDT/chemotherapy is consequently achieved. The polymeric prodrug PMP was synthesized according to our previous report^45^, and the partial characterization is shown in Supplementary Figures 1 and 2. PM and PMP polymeric micelles were prepared by dialysis method, and the critical micelles concentrations (CMC) of PM and PMP were 13.7 and 7.9 mg L^−1^, respectively, determined by fluorescence measurement using pyrene as a probe (Table 1). Dynamic light scattering (DLS) measurement showed that the hydrodynamic sizes of PM and PMP micelles were 37.8 ± 1.6 nm (PDI = 0.105 ± 0.012) and 63.2 ± 0.4 nm (PDI = 0.057 ± 0.009), respectively (Table 1, Fig. 2a). In addition, TEM images confirmed PM and PMP micelles were generally in spherical shape with a clear boundary (Supplementary Figures 3a, b). As compared with DLS results, the sizes of PM and PMP micelles obtained from TEM are smaller, probably due to the shrinkage of PEG shells upon drying. As we all known that the disulfide bond keeps stable under the normal physiological condition but could be cracked under the reducing condition. The effect of dithiothreitol (DTT) which mimics intracellular reducing conditions of tumor cells on the release of paclitaxel from PMP micelles was evaluated in aqueous solution. In contrast, paclitaxel was released from PMP micelles with a higher rate in the presence of 10 mM DTT within 48 h (Fig. 2b). For example, only less than 5% paclitaxel was released in PBS without DTT, while the release percentage of paclitaxel increased to nearly 70% in the presence of 10 mM DTT within 48 h. The results indicated that undesired leakage could be suppressed in normal physiological environment and that on-demand drug release capacity of PMP micelles could be achieved under the reducing condition. The spectra of ^1^H NMR and ESI-MS confirmed the AIEgen photosensitizer molecule 2,6-Bis(4-(diphenylamino)phenyl)-4,8-bis((2-ethylhexyl)oxy)benzo[1,2-b:4,5-b′]dithiophene 1,1,5,5-tetraoxide (TPA-BDTO, TB) was purposely synthesized according to our previous report (Supplementary Figures 4 and 5)^21^. The TB@PM and TB@PMP micelles were prepared by dialysis. As shown in Fig. 2a and Table 1, the hydrodynamic sizes of TB loaded polymer micelles (TB@PM and TB@PMP) were 60.2 ± 1.5 nm (PDI = 0.121 ± 0.016) and 73.4 ± 2.3 nm (PDI = 0.143 ± 0.015), respectively. After encapsulation of TB, TB@PM, and TB@PMP micelles became larger in size than the corresponding TB-free PM and PMP micelles, respectively. TEM image confirmed that TB@PM and TB@PMP aggregated into approximately spherical micelles with a clear boundary (Supplementary Figure 3C and D). Furthermore, the hydrodynamic size of TB@PMP micelles almost kept the same during a week, revealing that the prepared TB@PMP micelles possess good stability at room temperature (Fig. 2c). As shown in Supplementary Figure 6 and Supplementary Figure 3D, after 7-day storage, the shape and size of TB@PMP micelles were similar to the state 7 days ago, further proved that TB@PMP micelles possess good stability. TB exhibited a strong absorption peak at 530 nm in THF (Supplementary Figure 7A). TB@PMP micelles in aqueous solution showed two sharp absorption peaks at 232 and 530 nm, which were associated with paclitaxel and TB, respectively, and displayed a strong fluorescent peak at 684 nm from TB (Supplementary Figure 7B and C). The effect of reduction-sensitive property of TB@PMP micelles on TB fluorescent intensity was studied by monitoring change of fluorescent intensity in response to 10 mM DTT in aqueous solution. Also, there was no obvious decrease in fluorescent intensity during 30 h under reducing conditions of 10 mM DTT (Supplementary Figure 8), indicating that TB@PMP micelles could be used in bio-imaging with a high resistance to the reducing environments as the hydrophobic TB still maintained the aggregation state in aqueous solution.

**Table 1:** Properties of PM, PMP, TB@PM, and TB@PMP micelles

Samples	Size (nm)	PDI	CMC (mg L^−1^)	DLC (wt%)		Mass rationpaclitaxel: TB
				Paclitaxel	TB	
PM	37.8 ± 1.6	0.105 ± 0.012	13.7	/^b^	/^c^	/^d^
PMP	63.2 ± 0.4	0.057 ± 0.009	7.9	19.1	/^c^	/^d^
TB@PM	60.2 ± 1.5	0.121 ± 0.016	/^a^	/^b^	7.25	/^d^
TB@PMP	73.4 ± 2.3	0.143 ± 0.015	/^a^	17.3	9.34	1.85

^a^The concentration of PM and PMP higher than 13.7 and 7.9 mg L^−1^ could self-assemble into micelles in aqueous solution, respectively, and these micelles can subsequently load hydrophobic TB

^b^No paclitaxel was conjugated onto both PM and TB@PM micelles

^c^No TB was encapsulated into both PM and PMP micelles

^d^Paclitaxel and TB do not coexist in the PM, PMP, and TB@PM micelles

In addition, analysis of the released products from TB@PMP micelles in 10 mM DTT was conducted by liquid chromatography/high-resolution mass spectrometry. The mass spectrum showed that the disulfide bond was cleaved by DTT, which induced the breakdown of the neighboring ester bond to generate native paclitaxel (Fig. 2d). Also, the peak of TB was observed, which further indicated that the hydrophobic AIEgen photosensitizer could be successfully encapsulated into the hydrophobic core of micelles (Fig. 2e). The loading contents of paclitaxel and TB in TB@PMP micelles determined by UV–vis spectrophotometer were 17.3% and 9.34%, respectively (Table 1). The ROS generation capability of TB@PMP micelles was investigated using 9,10-anthracenediylbis(methylene)dimalonic acid as probe. As shown in Fig. 2f, the characteristic peak (400 nm) of anthracene moiety in 9,10-anthracenediylbis(methylene)dimalonic acid decreased to 20% of its original intensity after 20 min of light irradiation (white light, 100 mW cm^−2^), confirming the ROS generation of TB@PMP micelles with 10 mM DTT incubated at 37 °C for 0.5 h. The above results indicated that polymeric prodrug micelles TB@PMP possessed excellent paclitaxel and TB loading capacity as well as satisfactory ROS generation efficiency.

### Photostability and ROS generation studies of TB@PMP micelles in living cells

**Fig. 3:**
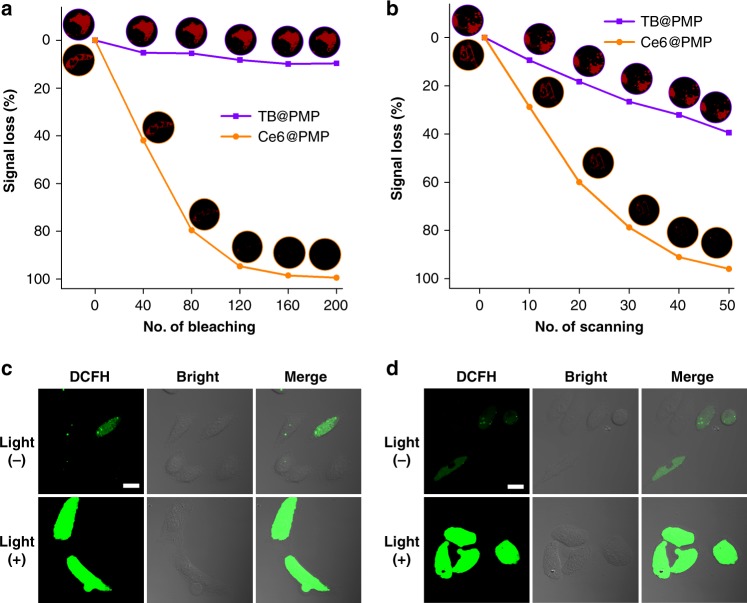
Photostability and ROS generation studies of TB@PMP micelles in living cells. **a** Signal loss (%) of fluorescent emission of TB@PMP and Ce6@PMP micelles with the increasing number of bleaching. Inset: corresponding CLSM images of HeLa cells. Excitation wavelength: 488 nm for TB@PMP and Ce6@PMP, 2.05 μs per pixel, irradiation time: 5.03 s bleaching^−1^; bleaching wavelength: 488 nm; bleaching intensity: 50%. **b** Signal loss (%) of fluorescent emission of TB@PMP and Ce6@PMP micelles with increasing number of scans. Inset: corresponding CLSM images of HeLa cells. Excitation wavelength: 488 nm for TB@PMP and Ce6@PMP, 4.01 μs per pixel, irradiation time: 40.27 s scan^−1^; scan wavelength: 488 nm; scan intensity: 8.6%. **c**, **d** Detection of intracellular ROS production by DCFH-DA in HeLa cells after incubation with TB@PM micelles and TB@PMP micelles, respectively, with or without light irradiation (white light, 100 mW cm^−2^, 3 min). Scale bar: 20 μm

Photostability is an important property to track the drug carriers in vivo, and the fluorescence signal was further used to produce ROS for image-guided PDT, which had irreversible damage to tumor cells and causes cell death^30^. Effective uptake by the tumor cells is one of the priority characteristics for these therapeutic micelles. First, TB@PM and TB@PMP micelles were incubated with HeLa cells for 4 h, respectively. As illustrated in Supplementary Figure 9, bright red fluorescence of TB loaded micelles was observed in the cytoplasm, demonstrating TB@PM and TB@PMP micelles had entered HeLa cells and mainly concentrated in the cytoplasm. Normally, macromolecule-based micelles can be internalized into cells mainly by pinocytosis. Pinocytosis can be further divided into clathrin-mediated endocytosis, macropinocytosis, and caveolae-mediated endocytosis. To identify specific internalization pathway, chlorpromazine, amiloride, and genistein were used as inhibitors for clathrin-mediated endocytosis, macropinocytosis, and caveolin-mediated endocytosis, respectively. As shown in Supplementary Figure 10, in the presence of chlorpromazine and genistein, the uptake of TB@PMP micelles was blocked but unaffected by amiloride. The inhibition study indicated that the cellular uptake of TB@PMP micelles is achieved by caveolae-mediated endocytosis and clathrin-mediated endocytosis, and clathrin-mediated endocytosis is the primary endocytosis pathway. In addition, photostability is a crucial parameter for developing fluorescent bio-imaging agents because high photostability allows the imaging process to withstand high-intensity laser scanning and to last for a long period with attenuated photobleaching. Continuous high-intensity bleaching and scanning by confocal laser scanning microscopy (CLSM) was used to quantitatively analyze the photostability of TB@PMP. Traditional photosensitizer, Chlorin e6 (Ce6) was physically encapsulated into PMP micelles to obtain Ce6@PMP micelles which was used as negative control. Two dishes of HeLa cells were incubated with TB@PMP and Ce6@PMP micelles for 4 h, respectively. As shown in Fig. 3a, the signal loss was <10% for TB@PMP micelles during 200 bleaches (about 17 min) upon excitation at 488 nm (bleaching laser power: 50%). In contrast, more than 90% fluorescence signal loss was observed after the 120th bleaching for Ce6@PMP. Upon continuous excitation at 488 nm (scanning laser power: 8.6%) during for 50 scans (about 34 min), more than 60% fluorescence signal remained for TB@PMP micelles, while <5% fluorescence signal remaining was recorded for Ce6@PMP (Fig. 3b). The results indicated that compared to traditional photosensitizer-loaded micelles, the TB@PMP micelles had satisfactory photostability and in favor of image-guided PDT.

After confirming the photostability of TB@PMP micelles, the intracellular ROS productivity by TB@PMP micelles in HeLa cells upon light irradiation was then evaluated using 2′,7′-dichlorofluorescin diacetate (DCFH-DA) as ROS sensor. It was known that non-emissive DCFH-DA could be oxidized to 2′,7′-dichlorofluorescin (DCFH) with green fluorescence upon reaction with ROS. After incubated with TB@PMP micelles for 4 h and irradiated with white light (100 mW cm^−2^) for 3 min, HeLa cells were investigated using CLSM. As shown in Fig. 3c and Fig. 3d, remarkable green fluorescence was found in HeLa cells upon irradiation for both TB@PMP and TB@PM micelles. This is due to the DCFH-DA oxidation by ROS produced from TB@PMP and TB@PM micelles, respectively (Supplementary Figure 11A and B). While extremely weak green fluorescence was detected in cells without light irradiation. Then the intracellular ROS productivity by Ce6@PMP micelles in HeLa cells upon light irradiation was evaluated using DCFH-DA as ROS sensor. As shown in Supplementary Figure 11C, much weaker green fluorescence was detected for Ce6@PMP compared to TB@PM and TB@PMP micelles. It demonstrated that Ce6@PMP micelles are not as good as TB@PM and TB@PMP micelles for a PDT agent. All the above results indicated that the AIEgen photosensitizer-loaded micelles can be used for image-guided PDT because of ROS productivity as well as satisfactory photostability in living cells.

### Chemotherapy and PDT-mediated apoptosis assay in living cells

**Fig. 4:**
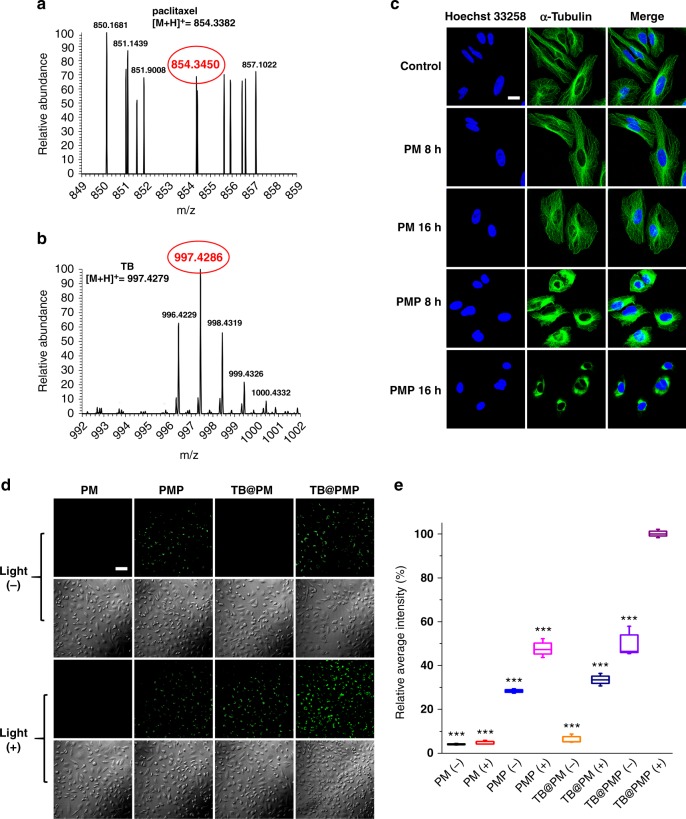
PDT and chemotherapy-mediated apoptosis assay in living cells. **a**, **b** Mass spectrum of the released products from HeLa cells incubated with TB@PMP micelles for 12 h. Mass spectrum of the released products from TB@PMP micelles with the treatment of 10 mM DTT for 12 h. HRMS (paclitaxel): [M+H]^+^ 854.3450 (calcd 854.3382); TB: [M+H]^+^ 997.4286 (calcd 997.4279). **c** Detecion of microtubules in HeLa cells after incubated with PM and PMP micelles for 8 and 16 h, respectively. Scale bar is 20 μm. **d** Cell apoptosis imaging using Annexin V-FITC in HeLa cells incubated with PM, PMP, TB@PM, and TB@PMP micelles with or without light irradiation (white light, 100 mW cm^−2^, 10 min) for 12 h, respectively. Scale bar is 20 μm. **e** Quantitative analysis of the fluorescence intensity in (**d**) by Image-Pro Plus (*n* = 3). Significant differences compared to the TB@PMP (+) group are indicated: ***p < 0.001

The effect on HeLa cells by chemotherapy and PDT-mediated apoptosis of PM, PMP, TB@PM, and TB@PMP micelles was studied. The therapy modality of PM, PMP, TB@PM, and TB@PMP micelles with or without light irradiation is listed in Table 1. PMP (−), PMP (+), and TB@PMP (−) were used as chemotherapy groups, and TB@PM (+) was used as PDT group. TB@PMP (+) was employed for combinational PDT/chemotherapy, while PM (−), PM (+), and TB@PM (−) were employed for negative controls. HeLa cells were incubated with TB@PMP micelles for 12 h, and then collected by centrifugation. Liquid chromatography/high-resolution mass spectrometry was used to analyze the cells lysate. As shown in Figs 4a, b, the peak of paclitaxel and TB was found, indicating that the native paclitaxel could release under the intracellular reducing conditions in tumor cells, and TB could be carried into cells by PMP micelles through hydrophobic effect.

As we know, paclitaxel is a hydrophobic drug that can promote the disassembly of microtubule and induce cell apoptosis^46^. Microtubules are natural biopolymers that continuously alter the state of their assembly and disassembly in seconds during the processes of most cellular activities^47^. To verify the effect of the reduction-sensitive paclitaxel-conjugated PMP micelles on microtubule integrity, the PMP micelles were incubated with HeLa cells and analyzed by CLSM. Both PM and PMP micelles were investigated after incubated with HeLa cells for 8 and 16 h, respectively. Microtubules were accurately identified by CLSM using anti-α-tubulin-FITC antibody as indicator (mouse monoclonal). As shown in Fig. 4c, the microtubules of control HeLa cells were outstretched and slender, exhibiting a well-organized cytoplasmic network. Compared to control cells, almost no obvious morphological change of microtubules was observed when incubated with PM micelles for both 8 and 16 h, respectively, indicating good biocompatibility of polycarbonate toward HeLa cells. However, it could be observed that the peripheral microtubules were shrunken moderately and damaged when HeLa cells were incubated with PMP micelles for 8 h. That was due to PMP that contains a reduction-sensitive paclitaxel polymeric prodrug. The released paclitaxel bound to a specific site of tubulin in cancer cells to prevent its depolymerization. As a result, the balance between microtubule aggregation and deaggregation was disrupted, causing the failed replication, and eventually leading to cancer cell apoptosis. Furthermore, the cells treated with PMP micelles for 16 h showed more condensed and enhanced damage and disruption to the microtubules compared with PMP micelles for 8 h (Fig. 4c). The results indicated that reduction-sensitive PMP micelles had an effective inhibitory effect on the microtubule of tumor cells. Besides, the PDT efficacy of TB loaded micelles was also analyzed by Calcein-AM staining assay which could identify live and apoptotic cells. As expected, strong green fluorescence was observed for PM (−) and PM (+) (white light, 100 mW cm^−2^, 20 min) without paclitaxel and TB, respectively (Supplementary Figure 12A), indicating negligible cell killing ability toward HeLa cells. In addition, the result of TB@PM (−) was similar to the PM micelles (Supplementary Figure 12A). As shown in Supplementary Figure 12B, the cells on the left of the dotted white line showed green fluorescence of Calcein for TB@PM micelles (−). While for TB@PM (+) (white light, 100 mW cm^−2^, 20 min) on the right of the dotted white line, there was almost no green fluorescence, which was probably owing to that HeLa cells were killed and washed away during the post-processing process. Thus, TB loaded micelles with satisfactory photostablility and ROS generation ability in vitro could serve as an effective platform for image-guided PDT against cancer cells.

Furthermore, the early apoptosis behaviors were in situ investigated by various samples using FITC-annexin V as the mediator. There was almost no green fluorescence observed for HeLa cells treated with PM (−) and PM (+), TB@PM (−), respectively, indicating negligible phototoxicity toward living cells. Then, almost the same green fluorescence intensity was observed in cells incubated with PMP (−), PMP (+), TB@PM (+), and TB@PMP (−), respectively. More interestingly, the green fluorescence intensity of cells treated with TB@PMP (+) was higher than other samples. These results clearly demonstrated the combinational therapy of PDT and chemotherapy possessed enhanced effect than chemotherapy or PDT only (Fig. 4d). Furthermore, the outcome of average intensity in Fig. 4e also verified the result of CLSM (Fig. 4d). The fluorescence intensity of TB@PMP (+) in cells was 2.9 and 2.6 times higher than the cells treated with PMP (−) and TB@PM (+), respectively. Based on the above-mentioned results, we can draw a conclusion that this novel combinational image-guided PDT/chemotherapy method exhibited an enhanced cell apoptosis than single-mode treatment (PDT or chemotherapy) ones.

### Cytotoxicity, combination index (CI) and cytotoxic mechanism studies in living cells

To get acquainted with the synergetic efficacy between PDT and chemotherapy, the cytotoxicity of prepared micelles with or without light irradiation against HeLa cells were studied by cell counting kit-8 (CCK-8) assay. As shown in Supplementary Figure 13A, the viability of cells to different concentrations of PM (−) for 48 h was over 90% in the range of 58.5–1000 μg mL^−1^. Besides, the impact of light irradiation (white light, 100 mW cm^−2^) for 20 min on cells was extremely weak (Supplementary Figure 13B). Negligible cytotoxicity was observed in TB@PM (−) incubated cells even in the high concentration of TB (16.2 μg mL^−1^) (Supplementary Figure 13C).

**Fig. 5:**
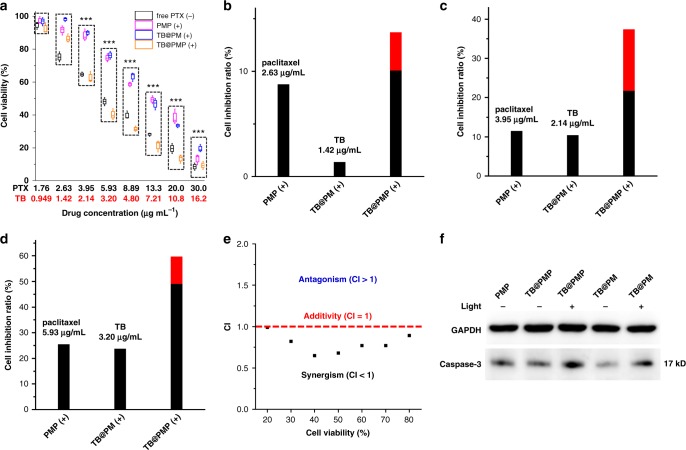
Cytotoxicity, combination index (CI) and caspas-3 expression studies in living cells. CCK-8 assay of free paclitaxel (−), PMP (+), TB@PM (+), and TB@PMP (+) (white light, 100 mW cm^−2^, 10 min) in HeLa cells after incubation for 48 h. The PMP (+) and TB @PM (+) groups showed significant differences compared to the TB @PMP (+) group: ***p < 0.001. **b**–**d** The inhibition ratios of PMP, TB@PM, and TB@PMP micelles treated cells upon light irradiation (white light, 100 mW cm^−2^, 10 min), respectively. The red bar denotes the additional cell inhibition ratio gained when TB@PMP micelles upon light irradiation are combined, compared with the sum of PMP, TB@PM micelles upon light irradiation. **e** Combination index (CI) plots TB@PMP (+) (white light, 100 mW cm^−2^, 10 min) treated HeLa cells at different cell viability from 20 to 80%. **f** Expressions of caspase-3 in HeLa cells treated with PMP (−), TB@PMP (−), TB@PMP (+), TB@PM (−), and TB@PMP (+) (white light, 100 mW cm^−2^, 10 min). And the full blot images are shown in Supplementary Figures 14B and C

As shown in Supplementary Figure 13D, PMP (−), PMP (+) (white light, 100 mW cm^−2^, 10 min) showed dose-dependent inhibition of cell proliferation in HeLa cells. However, there was no significant cytotoxicity difference between PMP (−) and PMP (+) toward HeLa cells, and the half maximal inhibitory concentration (IC_50_) value of PMP (−) and PMP (+) were 12.3 and 13.1 μg mL^−1^, respectively. This illustrated that the cell viability of PMP micelles without TB was unaffected by light irradiation. As shown in Fig. 5a, all the samples showed dose-dependent inhibition of cell proliferation in HeLa cells. First, free paclitaxel (−) exhibited more efficient cell-growth inhibition than PMP (+). The half maximal inhibitory concentration (IC_50_) value of free paclitaxel was 7.03 μg mL^−1^, while the IC_50_ value of PMP (+) was 13.1 μg mL^−1^. Next, we evaluated the cytotoxicity of PMP (+), TB@PM (+), and TB@PMP ( +) toward HeLa cells, which can be used for chemotherapy, PDT, and combinational PDT/chemotherapy, respectively. TB@PM (+) demonstrated a much higher cancer cell killing efficiency compared to that of TB@PM (−) (Fig. 5a and S15C). When paclitaxel was added, TB@PMP (+) (white light, 100 mW cm^−2^, 10 min) exhibited an enhanced cell-growth inhibition compared to PMP (+) and TB@PM (+) (Fig. 5a), revealing the successful therapeutic effect of this co-delivery micelles system. Furthermore, we found that the cell inhibition ratio was greater in combination therapy group (TB@PMP (+) (2.63 μg mL^−1^ of paclitaxel, 1.42 μg mL^−1^ of TB)) compared with the sum of each chemotherapy group (PMP (+)) and PDT group (TB@PM (+)) individually (Fig. 5b, red bar). The result was similar to the HeLa cells treated by PMP (+), TB@PM (+), and TB@PMP (+) with the other two concentrations (Fig. 5c, d, red bar). Generally, the combination index (CI) analysis was used to evaluate the synergistic effect of co-delivery systems. The value of CI >1, = 1, or <1 represented antagonism, additive, and synergism for combinational therapy, respectively^48^. In the following, we utilized CI for quantitatively assessment of the combinational therapy effect toward polymeric prodrug PMP and TB. The CI values of TB@PMP micelles were <1 at different cell viability from 20 to 80% (Fig. 5e), which demonstrated the synergistic combination effect of TB@PMP micelles due to the co-delivery of polymeric prodrug paclitaxel and TB against HeLa cells.

To understand the cytotoxic mechanism directly, western blotting analysis was employed to demonstrate the expression of apoptotic regulators (caspase-3) protein during the PDT/chemothreapy. Glyceraldehyde-3-phosphate dehydrogenase (GAPDH) was used as an internal control. Its gray value remained basically unchanged. As shown in Supplementary Figure 14A, B and C, the gray values ratios of caspase-3/GAPDH in untreated cells, PM (−), and PM (+) (white light, 100 mW cm^−2^, 10 min) treated cells were about 0.194, 0.233, and 0.119, respectively. It indicated that the impact of light irradiation and polycarbonate endocytosis on cells was extremely weak. As shown in Fig. 5f, the relationship of gray value ratios of caspase-3/GAPDH in TB@PMP (+), PMP (−), TB@PMP (−), TB@PM (+), and TB@PM (−) treated cells were about 0.713, 0.482, 0.418, 0.476, and 0.266, respectively. Obviously, TB@PMP (+) treated cells exhibited the highest gray value ratio of caspase-3/GAPDH (0.713). It is demonstrated that the micelles with PDT/chemotherapy effect could increase the gray value ratio, due to the generation of ROS by TB and chemotherapy by paclitaxel, which mediated the cell, apoptosis and lead to increased caspase-3 protein. The western blotting analysis results were coincident with that of the cytotoxicity by CCK-8 assay (Fig. 5a). All these results demonstrated that the combination of PDT and chemotherapy had a synergistic effect on HeLa cells indeed.

### In vivo tumor imaging and pharmacokinetic study

The hemolysis ratio was also evaluated by the UV absorbance of hemoglobin. It had been reported that the hemolysis ratio should be below 5% if the materials could be applied to intravenous injection^49^. Supplementary Figure 15A and B exhibits the hemolysis ratio of PM, PMP, TB@PM, and TB@PMP micelles in dark with different concentrations. The hemolysis ratios were all below 4% at different concentrations ranging from 20 to 200 μg mL^−1^, which indicated the co-delivery system was suitable for intravenous injection.

**Fig. 6:**
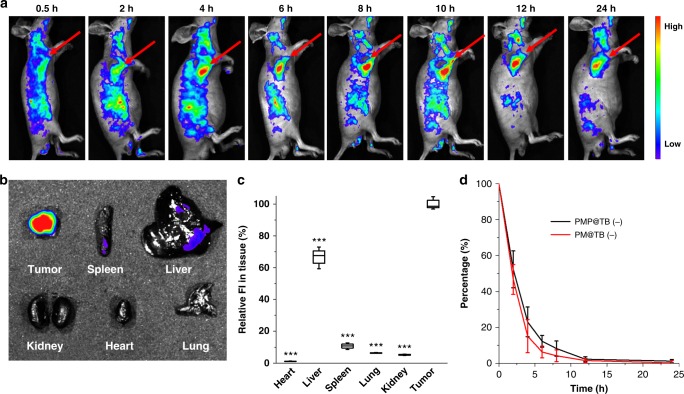
In vivo tumor imaging and pharmacokinetic study. **a** Optical imaging of HeLa tumor-bearing mice after the intravenous injection of TB@PMP micelles at different time. **b** Fluorescence images and **c** relative fluorescence intensity of the major organs and tumor of the TB@PMP micelles treated mice at 24 h postinjection (*n* = 3). Significant differences compared to the TB@PMP (+) group are indicated: ***p < 0.001. **d** The pharmacokinetics parameters of TB@PM and TB@PMP micelles (TB@PMP: 12.5 mg kg^−1^ body weight, an equal paclitaxel dose of 2.2 mg kg^−1^ and TB dose of 1.2 mg kg^−1^) after intravenous injection

Nanoparticles with the size ranging from about 5 to 200 nm are suitable for accumulation in tumor tissue in a passive manner via EPR effect after tail vein intravenous. As shown in Fig. 6a, TB@PMP micelles gradually accumulated in the tumor site, and the fluorescence intensity increased from 2 h after post tail vein intravenous and reached the maximum at 8 h. After 8 h, although the fluorescence intensity in tumor gradually decreased with time prolonging due to physiological metabolism, it was still observable even after 24 h. The PEG segment in TB@PMP micelles had played a crucial role in enhancing the biocompatibility and prolonging the half-life in blood^50^. Meanwhile, the tissue bio-distribution of TB@PMP micelles was performed at 24 h after postinjection by comparing the fluorescence in tumor and major organs. As shown in Fig. 6b, negligible fluorescence signal was observed in heart, spleen, lung, and kidney, weak fluorescence signal was founded in liver, and strong fluorescence signal was found in tumor tissue. The efficient and selective accumulation of TB@PMP micelles in tumor tissue could be attributed to EPR effect in a passive manner after post tail vein intravenous. Figure 6c showed the relative fluorescence intensity signal of TB@PMP micelles in tumor and organs, and the result was consistent with those observed in Fig. 6b. These results indicated that the TB@PMP micelles could selectively accumulate to tumor tissue. In the following, to investigate the elimination rate of TB@PM and TB@PMP micelles in blood, fixed volume of mice blood samples was obtained at predetermined time intervals. The change of fluorescence intensity was used to calculate the concentration of TB@PM and TB@PMP micelles in blood (Fig. 6d). In the whole, the above results verified that TB@PMP micelles could selectively accumulate in tumor tissue, which was favored for combinational image-guided PDT/chemotherapy in vivo.

### In vivo combinational therapy efficacy, systemic toxicity evaluation, imaging, and photostability

**Fig. 7:**
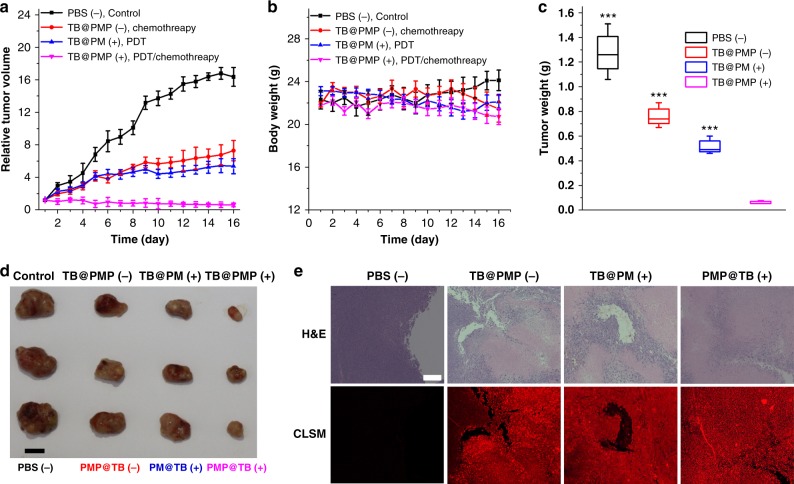
In vivo combinational therapy efficacy and imaging. **a** Relative tumor volume, **b** body weight, **c** tumor weight in the mice after the intravenous of different samples: PBS, TB@PM (+), TB@PMP (−), and TB@PMP (+) (532 nm, 250 mW cm^−2^ for 15 min) (*n* = 3). Significant differences compared to the TB@PMP (+) group are indicated: *p < 0.001. **d** Representative tumor images (scale bar: 1 cm). E) H&E staining and CLSM images of the different groups after 16 days intravenous injection treatment (scale bar: 10 μm)

The TB@PMP (−), TB@PM (+), and TB@PMP (+) (532 nm, 250 mW cm^−2^) possess the ability of chemotherapy, imaged-guided PDT, and combinational therapy (PDT/chemotherapy), respectively. The efficacy of these micelles was studied using HeLa-bearing mouse animal mode, and PBS (−) was used as negative control group. Light irradiation was performed after 8 h injection in order that micelles could be absorbed effectively by tumor tissue without apparent drug metabolism. As shown in Fig. 7a, compared with the mice group treated with PBS (−), after injecting TB@PMP (−) and PM@TB (+) for eight days continuously, the growth of tumor was inhibited, indicating the effective inhibition of tumor by paclitaxel and TB loaded micelles upon light irradiation, respectively. In sharp contrast, the mice group treated with TB@PMP (+) suppressed the tumor growth more remarkably than TB@PMP (−) and PM@TB (+). Meanwhile, negligible change in mice body weight was also observed in each group during the 16 days therapy (Fig. 7b), confirming that there was no excessive toxicity in each group. Remarkable differences in tumor weights between the mice group treated with TB@PMP (+) and other groups (Fig. 7c), indicated enhanced therapeutic effect of combinational image-guided PDT/chemotherapy in vivo, which was also visually verified by the representative tumor images in Fig. 7d.

Furthermore, an optical image of hematoxylin and eosin (H&E) stained tumor tissues at the 16th day for each group were also carried out (Supplementary Figure 16). Tumor tissue of the mice group treated with PBS (−) was compact with few apoptotic or necrotic. Tumor tissue of the mice group treated with TB@PMP (+) was observed sparser than TB@PMP (−) and TB@PM (+) treated ones, indicating more seriously apoptotic or necrotic of tumor tissue in combinational therapy group. The main organs were also analyzed by H&E staining. There were no obvious physiological morphology abnormalities in heart, liver, spleen, lung, and kidney for each group, confirming the low systemic toxicity for these samples.

Frozen sections of tumor tissue were observed under the CLSM for the detailed distribution of TB in tumor. As shown in Fig. 7e, red fluorescence intensity was barely observed in PBS (−) group, while high fluorescence was observed in TB@PMP (−), TB@PM (+), and TB@PMP (+) treated tumor tissue (Fig. 7e), which indicated that the TB loaded micelles could preferentially accumulate in the tumor tissue by EPR effect for image-guided PDT. Also, the photostability was studied for the frozen section of TB@PMP (+) group treated tumor tissue. These results (Supplementary Figure 17A and B) indicated that the TB@PMP micelles had appreciated photostability and were promising in image-guided PDT.

## Discussion

In summary, we described a reduction-sensitive co-delivery system based on polymeric prodrug micelles for combinational image-guided PDT/chemotherapy. Due to the high fluorescent efficiency in aggregate state of AIEgen photosensitizer, TB@PMP micelles overcome the limitations of many traditional photosensitizer and photosensitizer-loaded nanoparticles. Besides, TB@PMP micelles exhibit synergistic enhancement effect of PDT/chemotherapy upon light irradiation by the co-delivery of polymeric prodrug paclitaxel and TB against HeLa cells. In the HeLa cell tumor-bearing nude mouse model, the TB@PMP micelles with suitable particle size preferentially accumulate in the tumor tissue by EPR effect after intravenous injection, although the surface of the micelle has not been modified with active target functional groups. The image-guided PDT/chemotherapy of TB@PMP micelles upon light irradiation exhibits enhanced inhibition of the tumor growth compared to chemotherapy or PDT only. The results suggest a promising potential of the versatile AIEgen photosensitizer-loaded prodrug NPs for tumor-targeted imaging and combination therapy of TB and paclitaxel for other kinds of solid tumors. Introducing targeting ligands to the surfaces of TB@PMP micelles, such as antibodies, saccharides, and peptides, may further enhance the specificity. In addition, the AIEgen (TB) has bright two-photon fluorescence in aqueous solution.

## Methods

### Materials

Detailed material information was provided in our previous article^45^. Additionally, FITC Phalloidin, Calcein-AM, 2′,7′-dichlorofluorescin diacetate (DCFH-DA), and Annexin V-FITC were provided by yeasen Co. Ltd. (Shanghai, China). 9,10-anthracenediylbis(methylene)dimalonic acid (ABDA) was provided by Sigma-Aldrich.

### Cell culture

HeLa cells were cultured in DMEM medium in an atmosphere of 5% CO_2_ at 37 °C. The medium contained 1% antibiotics (penicillin-streptomycin, 10,000 U mL^−1^) and 10% heat-inactivated FBS.

### Synthesis of PEG-*b*-PMPMC-*g*-PTX (PMP)

PTX-SS-N_3_, PEG-*b*-PMPMC (PM), and PEG-*b*-PMPMC-*g*-PTX (PMP) were synthesized according to our previous literature^1^. PEG-*b*-PMPMC-*g*-PTX was synthesized by the conjugation of PTX-SS-N_3_ to the backbone of PEG-*b*-PMPMC through azide-alkyne CuAAC click reaction. The crude product was first purified by silica gel chromatography to remove copper and then precipitated into diethyl ether three times. Finally, the product was further purified by dialysis (cutoff Mw 3500) against DMSO for 48 h.

### 2,6-Bis(4-(diphenylamino)phenyl)-4,8-bis((2-ethylhexyl)oxy)benzo[1,2-b:4,5-b′]dithiophene 1,1,5,5-tetraoxide (TB)

According to a literature protocol^21^, 2,6-dibromo-4,8-bis((2-ethylhexyl)oxy)benzo[1,2-b:4,5-b′]dithiophene 1,1,5,5-tetraoxide (DBr-BDTO) (670 mg, 1 mmol), (4-(diphenylamino)phenyl)boronic acid (1150 mg, 4 mmol), tetrakis(triphenylphosphine)palladium (0) (58 mg, 0.05 mmol), and potassium carbonate (553 mg, 4 mmol) were added into 150 mL of 8:1:1 (v/v/v) mixture of toluene/ethanol/water under nitrogen atmosphere and refluxed for 12 h. The contents could cool to room temperature and filtered to remove insoluble impurities. Then the mixture was poured into water and extracted with dichloromethane. The organic layers were combined, the crude product was washed with aqueous NaHCO_3_ and water for several times, and then purified by silica column. A red solid was obtained finally. The yield is 53.2%. ^1^H NMR (CDCl_3_, 400 MHz): *δ* = 7.66–7.64 (m, 4H), 7.33–7.29 (m, 8H), 7.23 (s, 2H), 7.16–7.01 (m, 16H), 4.40 (d, 4H), 1.88–1.82 (m, 2H), 1.65–1.49 (m, 8H), 1.39–1.33 (m, 8H), 1.01–0.98 (t, 6H), 0.93–0.89 (t, 6H). High-resolution mass spectrum (HRMS) (MALDI-TOF) (C_62_H_64_N_2_O_6_S_2_): *m*/*z* 996.4184 calcd [M]^+^ 996.4200.

### Preparation of PM and PMP micelles, and determination of CMC

PM and PMP micelles were prepared by dialysis method. In brief, 20 mg PM was dissolved in 5 mL of THF and then dialyzed against ultrapure water for 24 h to obtain the PM micelles. The PMP micelles are prepared in a similar way to PM micelles. CMC was determined using pyrene as a fluorescence probe. The polymer solutions with various concentrations were added into sample bottles, and the concentration of pyrene was fixed at 6 × 10^−7^ M. The fluorescence emission spectrum of the polymer solution was recorded from 350 to 450 nm at an excitation wavelength of 334 nm. From the pyrene emission spectra, the intensity ratio (*I*_394_/*I*_378_) was analyzed as a function of the polymer concentration. The CMC value was determined as the cross-point when extrapolating the intensity ratio at the polymer concentration regions.

### Preparation of TB@PM and TB@PMP micelles

The TB loaded PM micelles were prepared by dialysis method, which was performed in the dark. PM (20 mg) and TB (2.4 mg) were dissolved in 5 mL of THF and then gently stirred for 6 h. Then 15 mL ultrapure water was slowly added to the solution, and the mixture was stirred for 12 h. The solution was transferred into a dialysis tube (cutoff Mw 3500) and dialyzed against ultrapure water for 24 h. The ultrapure water was refreshed every 4 h to remove the THF. Finally, the micelles solution was passed through 0.45 μm pore-sized syringe filter to remove the residual TB. The TB@PMP micelles are prepared in a similar way to TB@PM micelles.

### Particle size measurement

Nano-ZS ZEN3690 (Malvern Instruments) was used to detect the particle size at 25 °C. Particle size was measured in aqueous solution. The concentration of micelles was 0.1 mg mL^−1^.

### Transmission electron microscope (TEM) observation

Morphology of various samples were observed by TEM (JEM-2100 microscope). The sample was stained with 0.3% (w/v) phosphotungstic acid solution. The concentration of micelles was 0.1 mg mL^−1^.

### The drug loading capacity of paclitaxel and TB

The drug loading capacity (DLC) of paclitaxel and TB in PMP and TB@PMP was determined by UV–Vis spectrophotometer. Briefly, 2 mg lyophilized nanoparticles were dissolved in THF. UV–Vis spectrophotometer was used to determine the DLC of these samples after shaken for 1 h.

DLC was calculated as follows:

DLC = (weight of loaded drug/total weight of polymer and loaded drug) × 100%

### The release profiles in aqueous solution

The release profiles of paclitaxel from PMP micelles were studied at 37 °C in PBS (10 mM, pH 7.4) containing 0.1% (w/v) Tween 80 with or without 10 mM DTT by dialysis method. At predetermined time intervals, 4 mL of release medium was replaced with an equal volume of fresh media, the release medium was freeze-dried to obtain the released paclitaxel. The concentration of paclitaxel was determined by UV–Vis spectrophotometer.

### Detection of ROS in solution

The ROS generation was studied by using ABDA as an indicator as the absorbance of ABDA decreases upon reaction with ROS. Fifteen microliters of ABDA solution (4.5 mg mL^−1^ in DMSO) was added to 30 μg mL^−1^ TB@PMP micelles. Afterward, 1.5 mL mixed solution in 5 mL EP tube was irradiated with light (white light, 100 mW cm^−2^). The decomposition of ABDA was monitored by the absorbance decrease. The absorbance decrease of ABDA at 400 nm was recorded for different durations of light irradiation to obtain the decay rate of the photosensitizing process.

### TB@PM and TB@PMP micelles phagocytosed by the cancer cells

HeLa cells were placed on microscope slides in cell culture medium incubated with TB@PM (137.9 μg mL^−1^) and TB@PMP micelles (107.1 μg mL^−1^) at 37 °C under 5% CO_2_ atmosphere, respectively. After incubation for 4 h, the culture medium was removed and the cells were washed three times with PBS. Then the cells were fixed with 4% paraformaldehyde and the nuclei were stained with Hoechst 33258. Blue fluorescence (nucleus dyed with Hoechst 33258, Ex: 405 nm, Em: 425-475 nm); red fluorescence (TB, Ex: 488 nm, Em: 620–720 nm).

### Photostability test

Firstly, cells were incubated with TB@PMP micelles and Ce6@PMP micelles, respectively. And then imaged by CLSM (Zeiss LSM 880) TB@PMP micelles were excited at 488 nm (50% laser power for bleaching, 8.6% laser power for scanning) and the fluorescence was collected at 620–720 nm. Ce6@PMP micelles were excited at 488 nm (50% laser power for bleaching, 8.6% laser power for scan) and fluorescence was collected in the range of 650–750 nm. The way of photostability test of tumor tissue is similar to the cells.

### Singlet oxygen detection in living cell

HeLa cells were incubated with TB@PM (137.9 μg mL^−1^) and TB@PMP micelles (107.1 μg mL^−1^) for 4 h, respectively. Then the medium was replaced. DCFH-DA was added (final concentration 1 × 10^−5^ M) and the cells were incubated for 20 min. Ten minutes light irradiation was performed subsequently (white light, 100 mW cm^−2^, 5 min). The cells were repeatedly washed and then observed as soon as possible via CLSM. Green fluorescence (DCFH, Ex: 488 nm, Em: 505–540 nm); red fluorescence (TB@PMP, Ex: 488 nm, Em: 620–720 nm), scale bar: 20 μm.

### Detection of microtubules in living cells

The cells were incubated with PM and PMP micelles in dark for 8 and 16 h, respectively. The culture medium was removed and washed thrice with PBS and fixed with 4% paraformaldehyde for 10 min. After permeabilized with 0.1% Triton X-100 in PBS for 10 min, cells were washed with PBS three times and stained with anti-α-tubulin-FITC at 37 °C in the dark for 1 h. Nuclei were stained with Hoechst 33258 for 15 min. Blue fluorescence (nucleus dyed with Hoechst 33258, Ex: 405 nm, Em: 425–475 nm), green fluorescence (anti-α-tubulin-FITC, Ex: 488 nm, Em: 505–540 nm), scale bar: 20 μm.

### Annexin V-FITC assay in vitro

HeLa cells were incubated with PM (127.9 μg mL^−1^), PMP (97.0 μg mL^−1^), TB@PM (137.9 μg mL^−1^), and TB@PMP micelles (107.1 μg mL^−1^) for 4 h, respectively. After washed three times using PBS, the cells were treated with light irradiation (white light, 100 mW cm^−2^ for 10 min), and then the cells were further cultured for 12 h and stained with Annexin V-FITC in binding buffer for 15 min before observed via CLSM. Green fluorescence (Annexin V-FITC, Ex: 488 nm, Em: 505–540 nm). Quantitative analysis of the FITC fluorescence intensity was done by Image-Pro Plus (Edit → Convert to → Gray Scale 16).

### PDT-mediated apoptosis assay

HeLa cells were incubated with PM (255.9 μg mL^−1^) and TB@PM micelles (275.9 μg mL^−1^) for 4 h. After washing three times using PBS, the cells were treated with light irradiation (white light, 100 mW cm^−2^ for 20 min), the area of illumination is controlled by masking. Then the cells were further cultured for 12 h. After this treatment, cells were stained with Calcein-AM for 15 min and washed seven times with fresh culture medium to remove dead cells and Calcein-AM before imaging (Calcein-AM, Ex: 488 nm, Em: 505–525 nm).

### Western blot analysis for caspase-3 expression

After incubated with various samples in HeLa cells for 24 h, cells were lysed with 50 μL RIPA buffer and resuspended in 50 μL 2 × SDS buffer containing 1% β-mercaptoethanol. Then the samples were heated for 5 min and separated on a 10% SDS-PAGE (15 μL per lane). After electrophoresis, proteins were transferred to a PVDF membrane (Millipore). The PVDF membranes were then blocked in PBS with 5% skim milk for 1 h. Cleaved caspase-3 was detected by incubating the membranes with the primary antibody rabbit anti-human caspase-3 (1:2000 dilution) overnight at 4 °C and then with the secondary antibody HRP labeled goat antirabbit IgG (1:3000 dilution, Google Biotechnology, Wuhan) for 1 h. Specific proteins were monitored by enhanced chemiluminescence. GAPDH was employed as protein loading control.

### Hemolysis test

The release of hemoglobin from mice blood cells was used to evaluate the hemolytic activities of PM, PMP, TB@PM, and TB@PMP micelles by spectrophotometry. The blood samples were centrifugated and resuspended in normal saline to get the red blood cells (RBCs 2%). 0.5 mL RBCs suspension mixed with 0.5 mL ultrapure water and 0.5 mL normal saline solution were served as positive control (producing 100% hemolysis) and negative control (producing no hemolysis), respectively. 0.5 mL of material solutions were added into the mixture of 0.5 mL RBCs suspension. After kept at 37 °C for 3 h, all the samples were centrifuged. The absorbance of supernatants was measured with UV spectrophotometer and the normal saline was used as blank. The hemolysis ratio of RBSs was calculated using the following formula: hemolysis (%) = (*A*_sample_ − *A*_negative_)/(*A*_positive_ − *A*_negative_) × 100%, where *A*_sample_, *A*_negative_, and *A*_positive_ refer to the absorption of material sample solution, negative control, and positive control at 570 nm, respectively.

### CCK-8 assay

The cytotoxicity assessment was carried out in HeLa cells by CCK-8. Briefly, 1 × 10^4^ of cell suspension were seeded into each well of a 96-well plate and incubated for 24 h. Then medium was replaced with samples at various concentrations, respectively. The culture medium was replaced with 200 μL of fresh medium at 4 h post incubation. Then part of samples was treated with light irradiation (white light, 100 mW cm^−2^, 10 min) and then further incubated for 48 h. After that, 10 μL of CCK-8 solution was added into each well and the cells were incubated for another 1 h. The absorbance at 450 nm was recorded. Cell viability was expressed by the ratio of the absorbance of CCK-8 in cells incubated with samples to that of the cells incubated with culture medium only.

### Animal model

Nude mice weighing 20 g were purchased from HFK Bioscience Co. (Beijing, China). All the animal studies were performed in compliance with guidelines set by the Animal Care Committee at Tongji Medical College. To establish the HeLa tumor model, 1 × 10^6^ cells were inoculated subcutaneously into the right front flanks of male BALB/c nude mice. Tumor growth was measured using a caliper, and the tumor volume was calculated using the following formula: volume = ((tumor length) × (tumor width)^2^)/2.

### Pharmacokinetic study

Blood samples of BALB/c mice were obtained at preset times after intravenous injection of TB@PM and TB@PMP micelles (2.0 mg mL^−1^ in PBS, 125 μL). The blood samples were then diluted with PBS and were repeatedly freeze-thawed. Subsequently, cells were under ultrasound for 5 min, the fluorescence intensities of samples were recorded at the excitation wavelength of 530 nm.

### In vivo tumor imaging and major organs distributions

When tumor volume reached around 100 mm^3^, male BALB/c with a HeLa tumor xenograft under the armpit were intravenously injected with TB@PMP micelles solutions (2.0 mg mL^−1^ in PBS, 125 μL) through tail vein. At predetermined time points (0.5, 2, 4, 6, 8, 10, 12, and 24 h postinjection), The mice were monitored by the IVIS Spectrum (PerkinElmer) (Ex = 535 nm, Em = 620–720 nm). At 24 h, the heart, liver, spleen, lung, kidney, and tumor tissue were collected and for tissue distribution study^51^.

### In vivo antitumor study by intravenous injection

Male BALB/c with a HeLa tumor xenograft under the armpit were randomly divided into four groups, and intravenously injected with PBS, TB@PM, TB@PM, and TB@PMP (2.0 mg mL^−1^, 125 μL for micelles) respectively on the first, fourth, seventh, tenth, and thirteenth days. Eight hours later, the tumors of mice of TB@PM (+) and TB@PMP (+) group exposed to 532 nm light irradiation (250 mW cm^−2^) for 15 min. The mice weight and tumor volume of each group were daily measured. Relative tumor volume was calculated as *V*/*V*_0_, *V*_0_ was the tumor volume on the first day before treatment. Meanwhile, the tumor, heart, liver, spleen, lung, and kidney of each group were collected and studied by H&E staining.

## Electronic supplementary material

Supplementary Information

## Data Availability

Data supporting the findings of this study are available within this article and its Supplementary Information file, and from the corresponding author on reasonable request.
