# Supplementary material for: A high therapeutic efficacy of polymeric prodrug nano-assembly for a combination of photodynamic therapy and chemotherapy
Source: Commun Biol. 2018 Nov 21;1:202. doi: 10.1038/s42003-018-0204-6 (PMC6249255; doi:10.1038/s42003-018-0204-6)
Supplement: Supplementary file 1 — Supplementary Information [file 42003_2018_204_MOESM1_ESM.pdf]

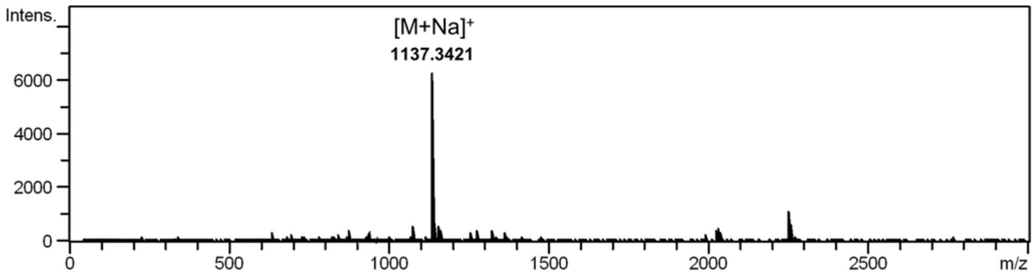

Supplementary Figure 1. High resolution mass spectrum (HRMS) (MALDI-TOF) spectrum of PTX-SS-N_3, calcd $[M+Na]^+$ 1137.3443.

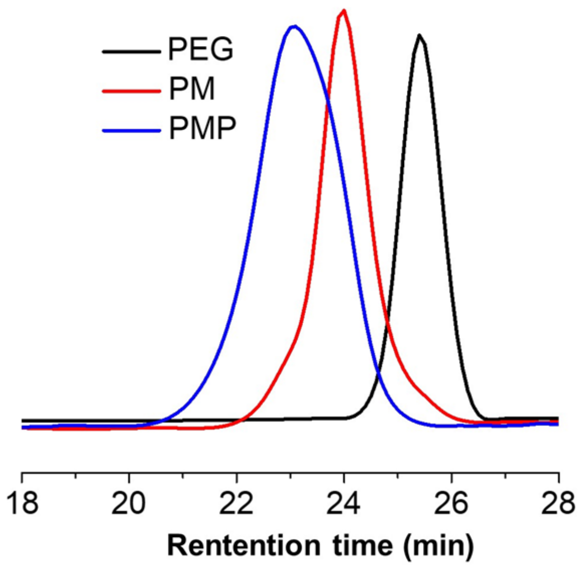

Supplementary Figure 2. Gel Permeation Chromatography (GPC) traces of PEG, PM and PMP.

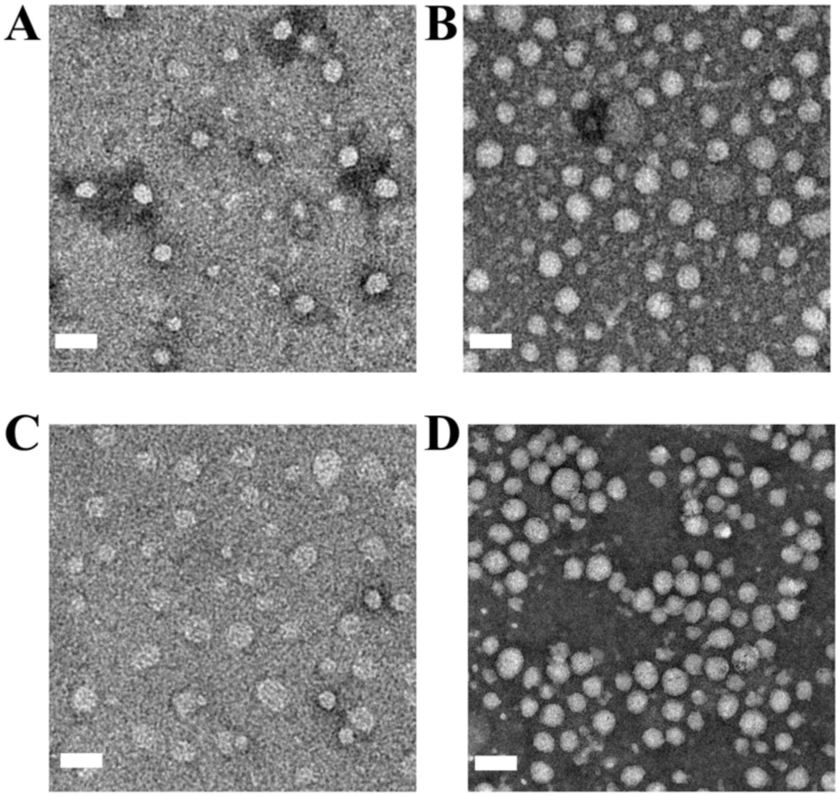

Supplementary Figure 3. The TEM image of **A)** PM, **B)** PMP, **C)** TB@PM and **D)** TB@PMP micelles, scale bar: 50 nm.

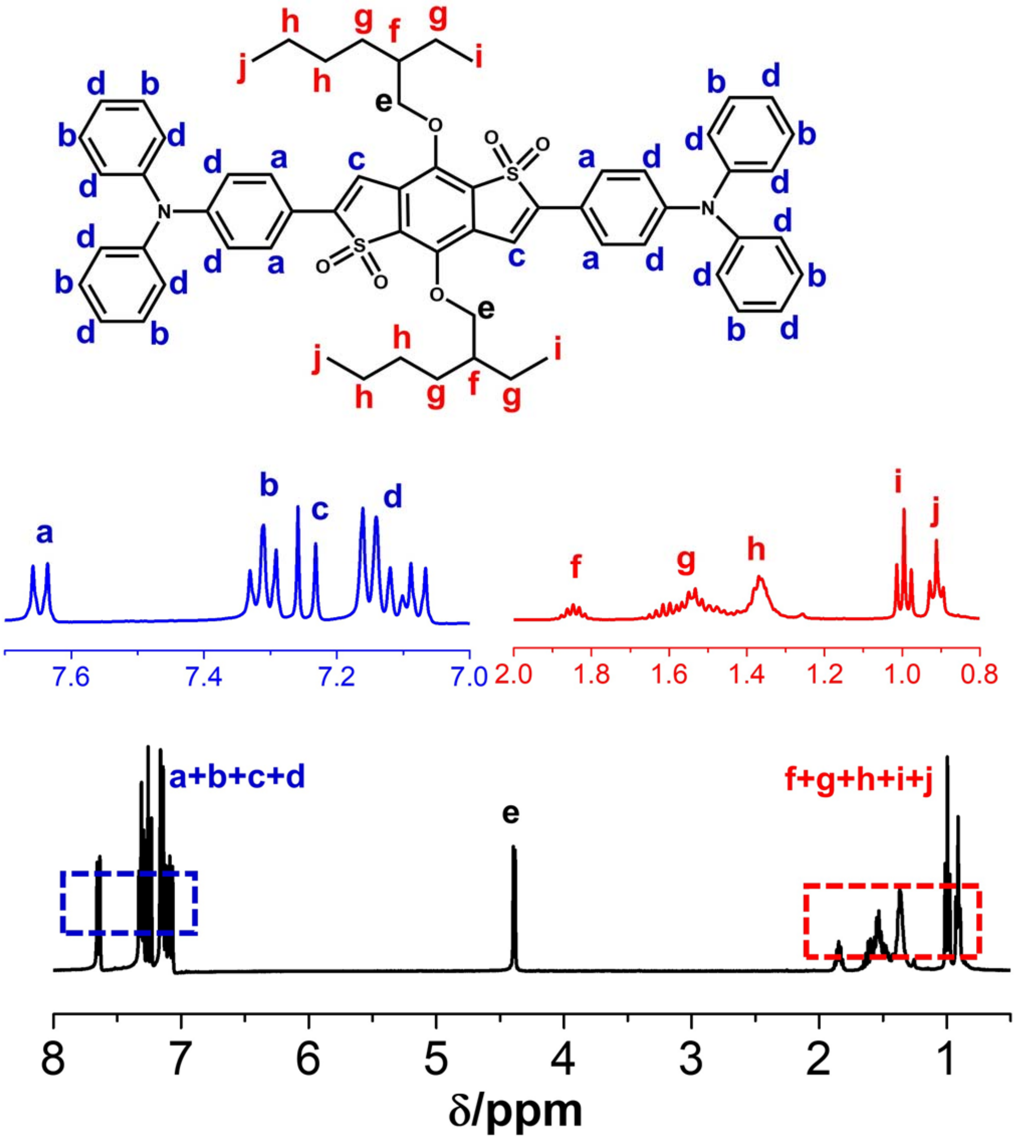

Supplementary Figure 4. Structure and ^{1}H NMR spectrum of TB.

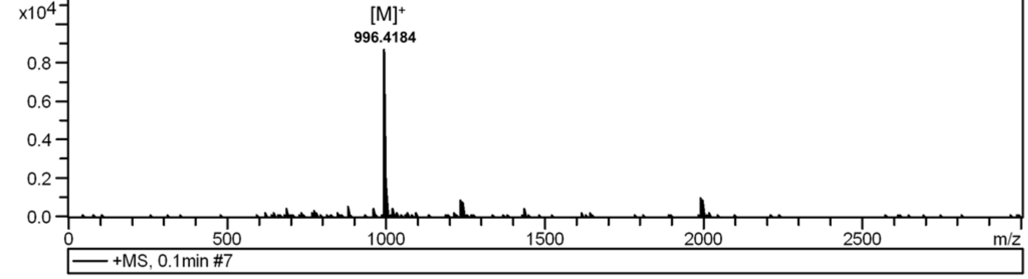

Supplementary Figure 5. HRMS (MALDI-TOF) spectrum of TB, calcd $[M]^+$ 996.4200.

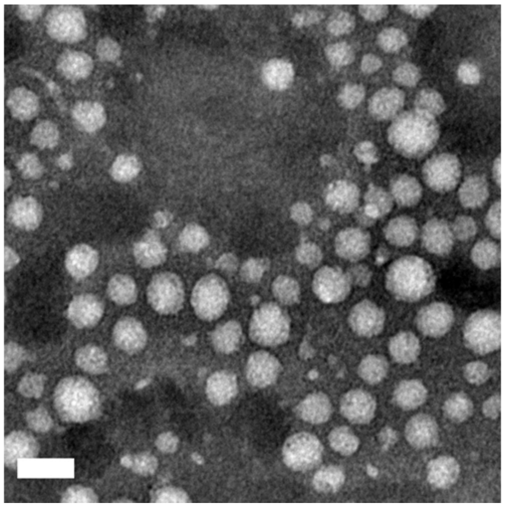

Supplementary Figure 6. The TEM image of TB@PMP micelles after 7 days storage at room temperature, scale bar: 50 nm.

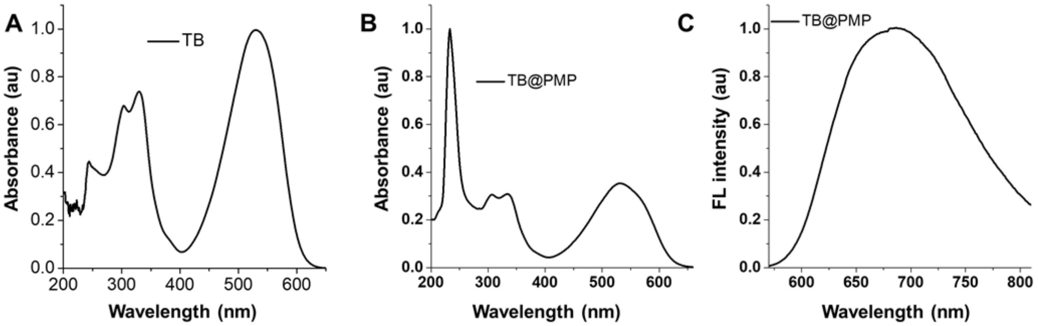

Supplementary Figure 7. A) Absorption spectrum of TB (2.8×10^{-6} M) in THF; **B)** Absorption of TB@PMP micelles (30 µg mL^{-}) in aqueous solution; **C)** FL spectra (Ex: 530 nm) of TB@PMP micelles (30 µg mL^{-}) in aqueous solution.

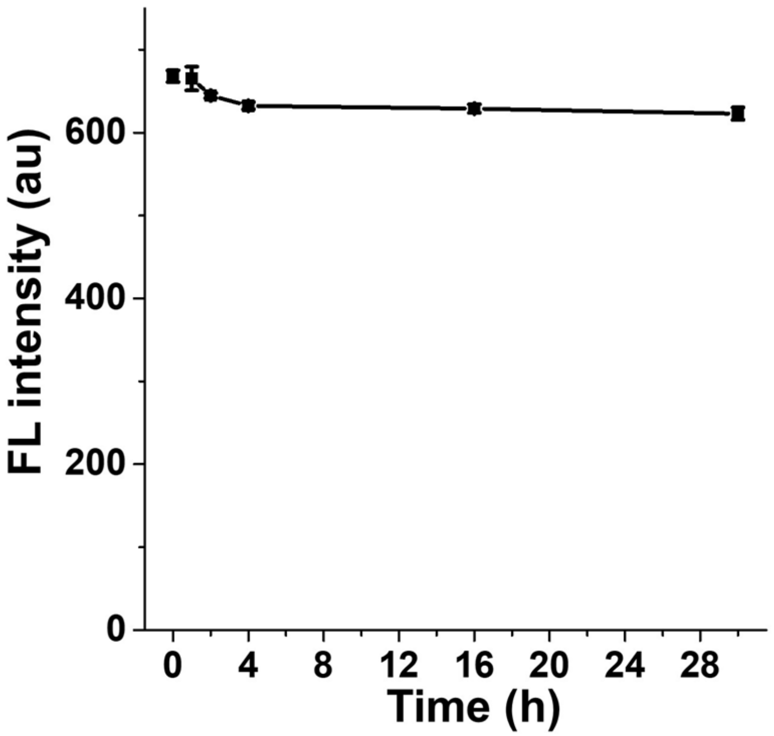

Supplementary Figure 8. Fluorescence intensity of 630 nm of TB@PMP micelles (30 μg mL^{-1}) in aqueous solution with 10 mM DTT (Ex: 530 nm).

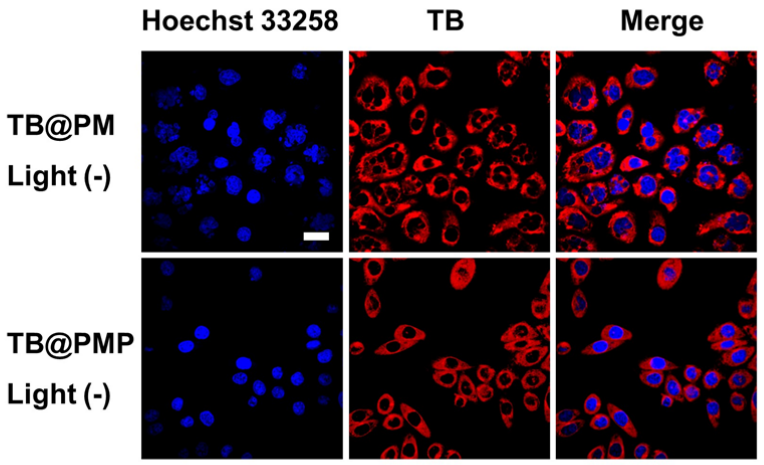

Supplementary Figure 9. Confocal images of HeLa cells incubated with TB@PMP and TB@PMP micelles (250 μg mL^{-1}) for 4 h in dark, respectively. Scale bar: 20 μm.

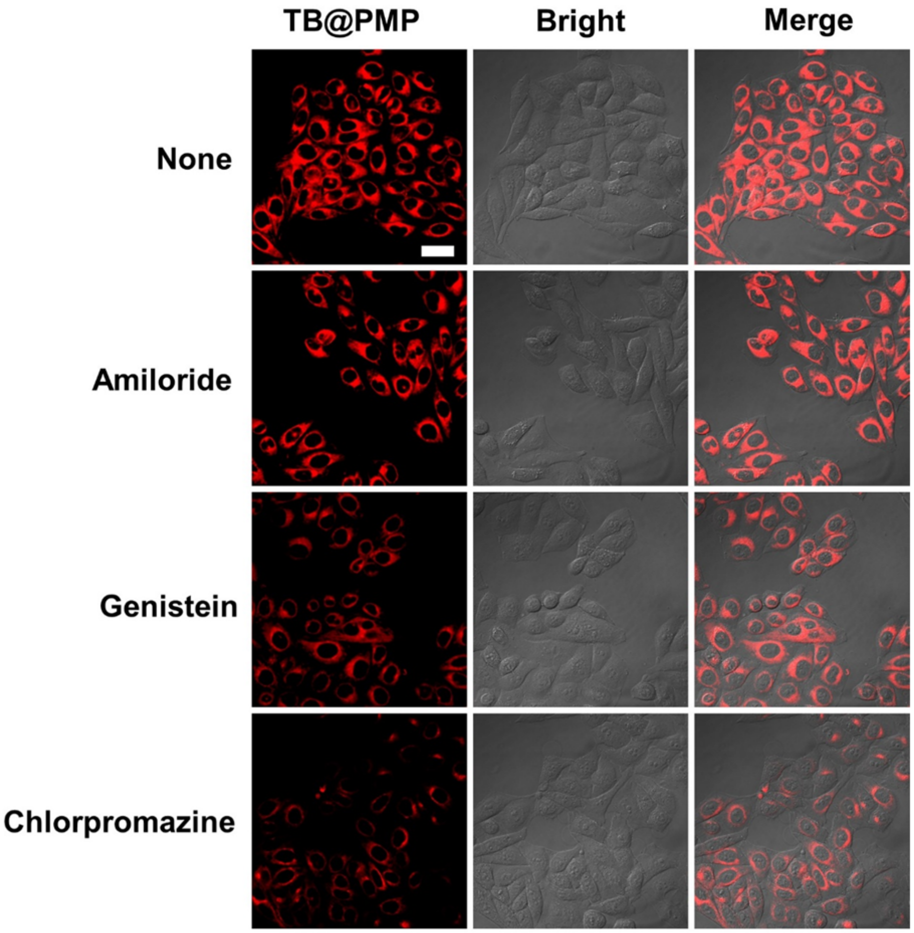

Supplementary Figure 10. Confocal laser scanning microscopy images of HeLa cells after incubation with TB@PMP micelles (250 μg mL^{-1}) containing different endocytosis inhibitor: none, Amiloride, genistein, and chlororomazine. Scale bar: 20 μm.

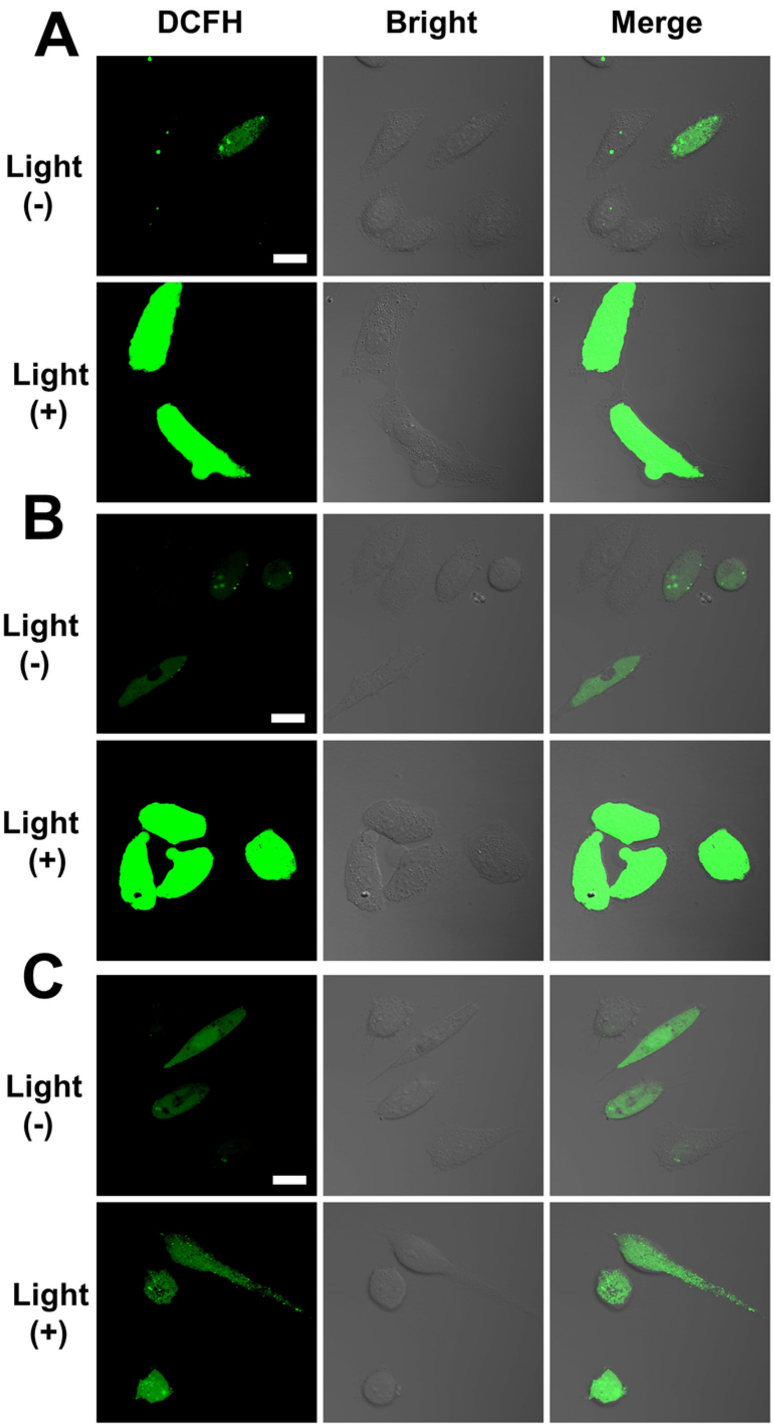

Supplementary Figure 11. Detection of intracellular ROS production by DCFH-DA in HeLa cells after incubation with TB@PM micelles (A), TB@PMP micelles (B) and Ce6@PMP micelles (C) respectively, without or with light irradiation (white light, 100 mW cm^{-2}, 3 min) (the parameters of green light channel of all samples were adjusted to a high level compared to Figure 3C and D). Scale bar: 20 μm.

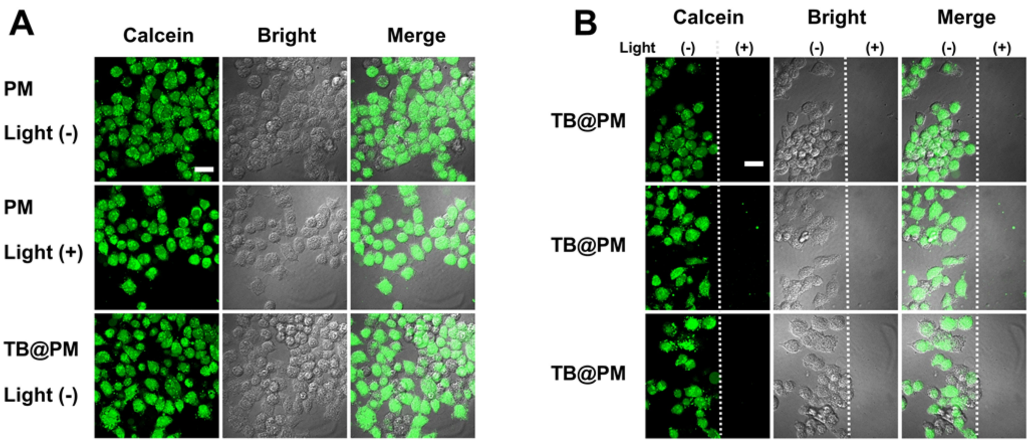

Supplementary Figure 12. A) and **B)** PM, TB@PM micelles were without or with light irradiation (white light, 100 mW cm^{-2}, 20 min) incubated with HeLa cells for 12 h. The cells on the left of the dotted white line were without light irradiation, and the right ones were with light irradiation. Scale bar: 20 μm.

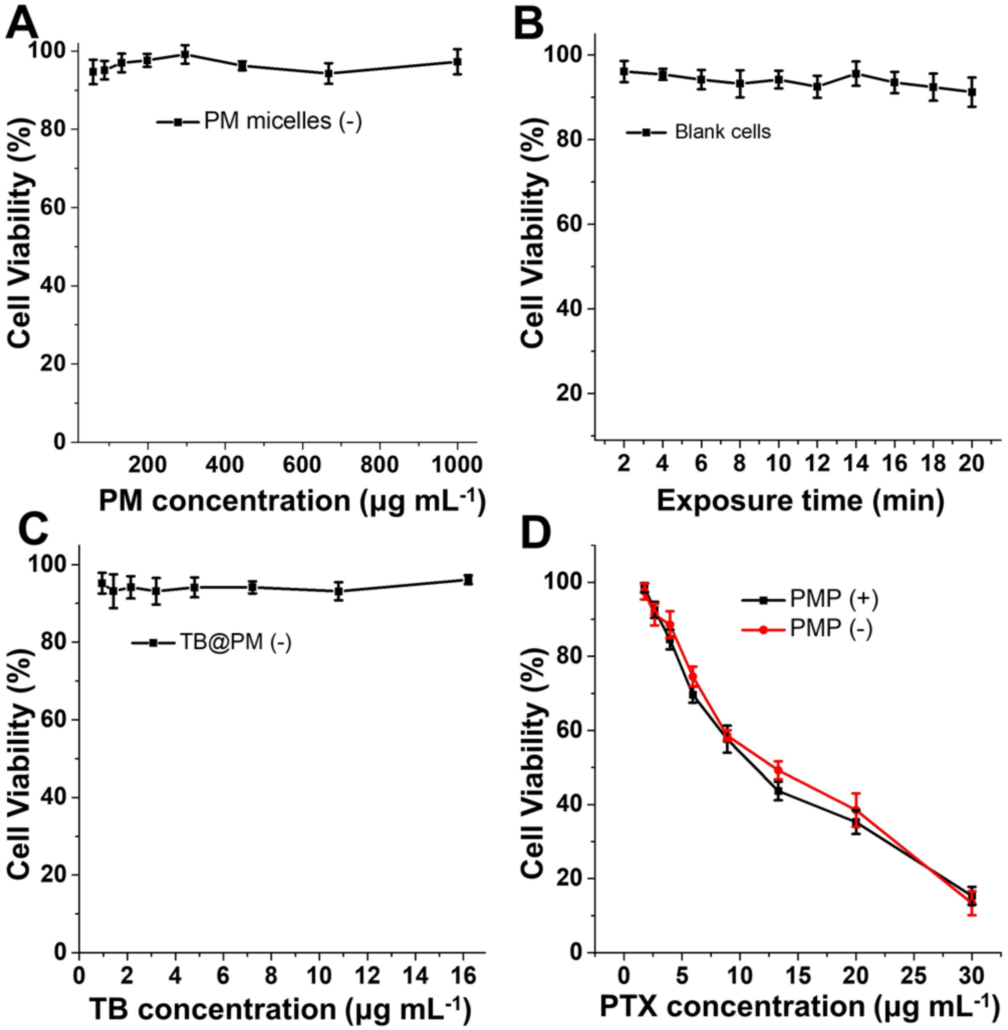

Supplementary Figure 13. CCK-8 assay of **A)** PM (-), **B)** different times at a power density of 100 mW cm^{-2}, **C)** TB@PM (-), and **D)** PMP (-), PMP (+) (white light, 100 mW cm^{-2}, 10 min) in HeLa cells after incubation for 48 h.

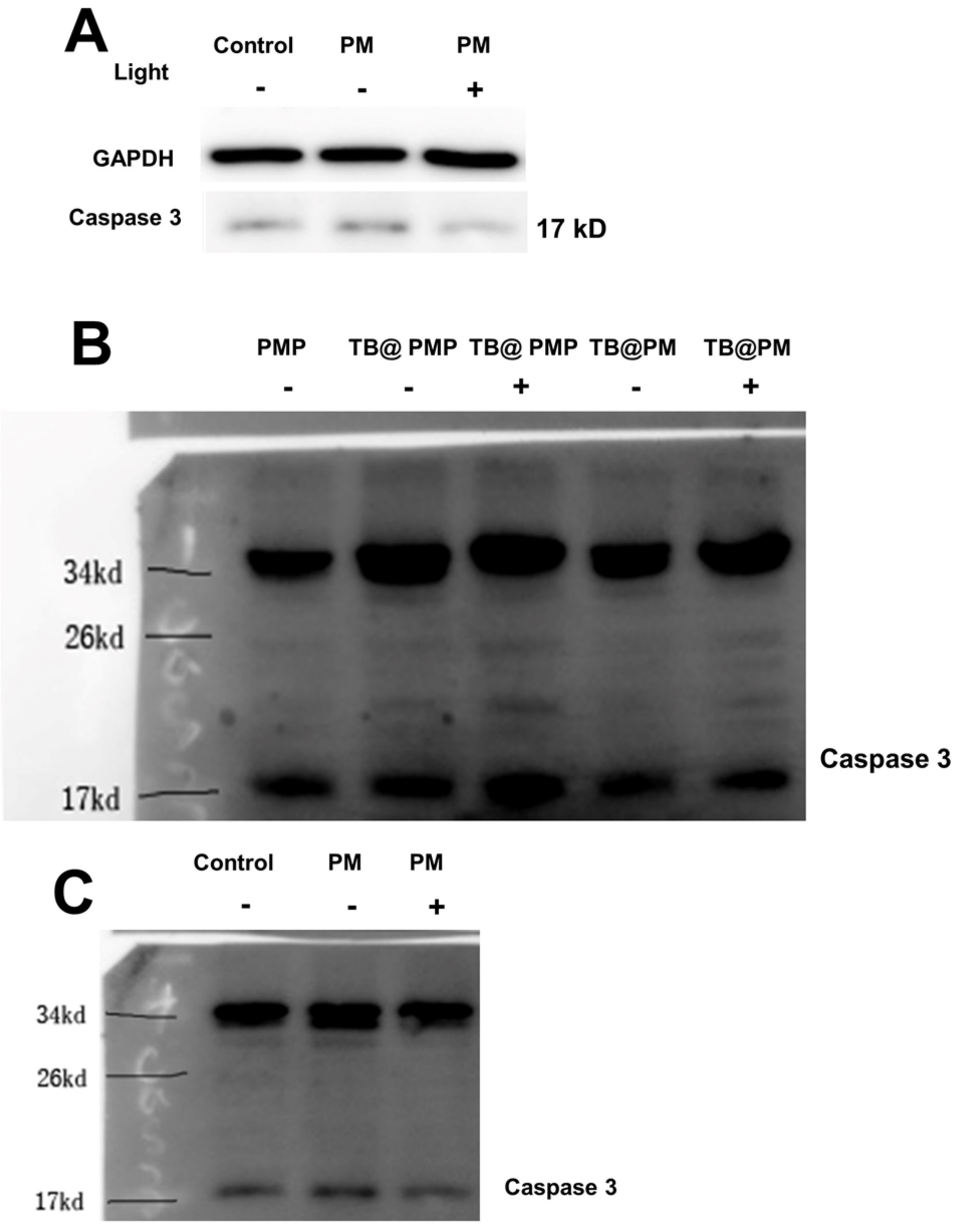

Supplementary Figure 14. A) Expressions of caspase 3 in HeLa cells of control group (-), PM (-), PM (+) (white light, 100 mW cm^{-2}, 10 min), GAPDH was used as a control; **B** and **C)** original blot images of caspase 3 in HeLa cells of Figure 5F and Supplementary Figure 14A, respectively.

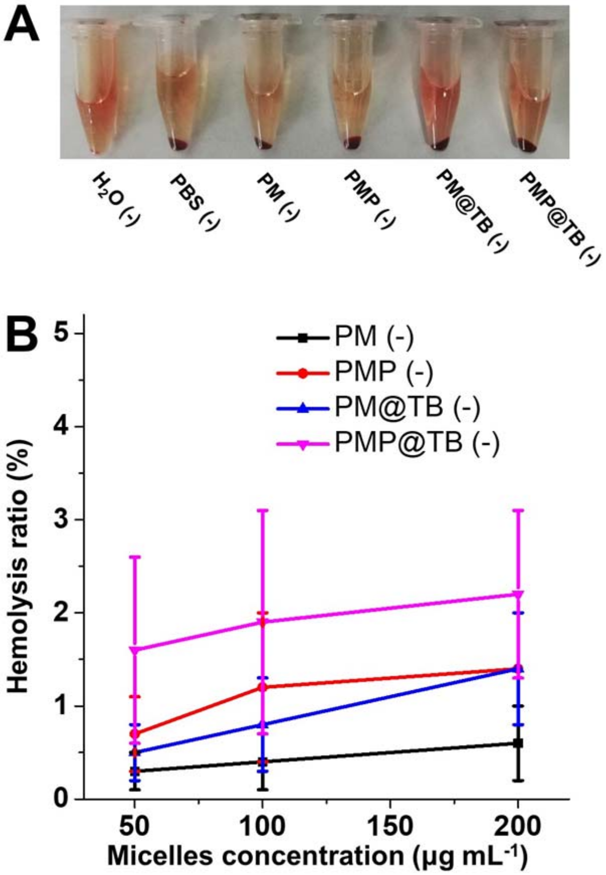

Supplementary Figure 15. A) Photographs of hemolysis assay after incubation with ultrapure water (positive control), PBS (negative control), and PM, PMP, TB@PM, and TB@PMP micelles for 3 h in dark. The presence of large amounts of hemoglobin in the supernatant is observed only in the positive control tub saline; **B)** The hemolysis ratio induced by PM, PMP, TB@PM, and TB@PMP micelles with different concentration incubated at 37 °C for 3 h in dark.

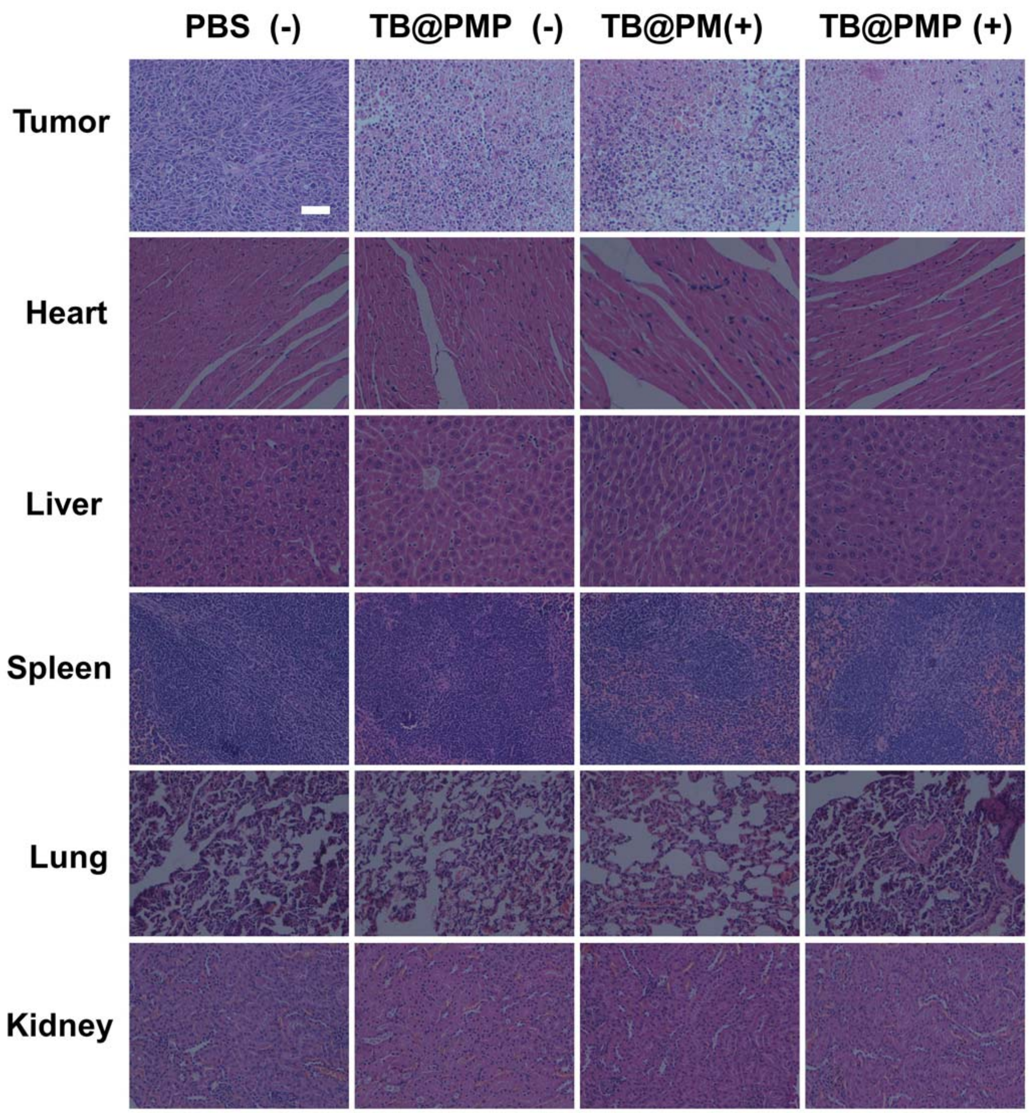

Supplementary Figure 16. H&E staining images of the tumor organs obtained from the mice after different treatments: intravenous injection of different samples: PBS, TB@PM, and TB@PMP micelles and light irradiation treatment or not (532 nm, 250 mW cm^{-2} for 30 min). Scale bar: 100 μm.

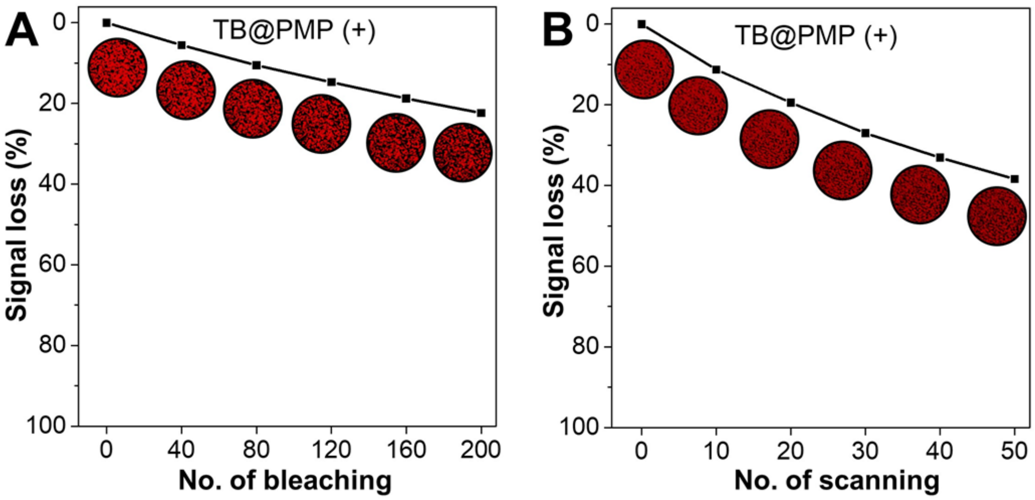

Supplementary Figure 17. A) Signal loss (%) of fluorescent emission of tumor tissue treatment by TB@PMP (+) with increasing number of bleaching. Inset: Corresponding CLSM images of tumor tissue. 2.05 μs per pixel, irradiation time: 6.201 s per bleaching; bleaching wavelength: 488 nm; bleaching intensity: 50%; **B)** Signal loss (%) of fluorescent emission of tumor tissue treatment by TB@PMP (+) with increasing number of scans. Inset: CLSM images of tumor tissue; 4.01 μs per pixel, irradiation time: 40.27 sper scan; scan wavelength: 488 nm; scan intensity: 8.6%.
